# Alzheimer’s Disease: What Can We Learn From the Peripheral Olfactory System?

**DOI:** 10.3389/fnins.2020.00440

**Published:** 2020-05-19

**Authors:** Michele Dibattista, Simone Pifferi, Anna Menini, Johannes Reisert

**Affiliations:** ^1^Department of Basic Medical Sciences, Neuroscience and Sensory Organs, University of Bari A. Moro, Bari, Italy; ^2^Neurobiology Group, SISSA, Scuola Internazionale Superiore di Studi Avanzati, Trieste, Italy; ^3^Monell Chemical Senses Center, Philadelphia, PA, United States

**Keywords:** olfaction, neurodegeneration, Alzheiemer’s disease, odorant receptor (OR), biomarkes

## Abstract

The sense of smell has been shown to deteriorate in patients with some neurodegenerative disorders. In Parkinson’s disease (PD) and Alzheimer’s disease (AD), decreased ability to smell is associated with early disease stages. Thus, olfactory neurons in the nose and olfactory bulb (OB) may provide a window into brain physiology and pathophysiology to address the pathogenesis of neurodegenerative diseases. Because nasal olfactory receptor neurons regenerate throughout life, the olfactory system offers a broad variety of cellular mechanisms that could be altered in AD, including odorant receptor expression, neurogenesis and neurodegeneration in the olfactory epithelium, axonal targeting to the OB, and synaptogenesis and neurogenesis in the OB. This review focuses on pathophysiological changes in the periphery of the olfactory system during the progression of AD in mice, highlighting how the olfactory epithelium and the OB are particularly sensitive to changes in proteins and enzymes involved in AD pathogenesis. Evidence reviewed here in the context of the emergence of other typical pathological changes in AD suggests that olfactory impairments could be used to understand the molecular mechanisms involved in the early phases of the pathology.

## Introduction

The sense of smell makes an important and often underestimated contribution to our quality of life. New evidence is increasingly debunking the old myth that in humans the sense of smell is scarcely sensitive and of limited use (McGann, 2017). Many of our daily actions are driven by olfaction (i.e., olfactory-driven food choice, detection of spoiled food before it enters the mouth).

The olfactory system has remarkable features unique among sensory systems in particular and the nervous system in general. It is the only sensory organ that has its primary neurons in direct contact with the external world: the olfactory receptor neurons (ORNs) in the nasal cavity. Olfactory receptor neurons are bipolar neurons that, throughout the life span of an animal, degenerate and are replaced by new neurons originating from a pool of stem cells in the basal part of the olfactory epithelium (OE; Kondo et al., 2010; Brann and Firestein, 2014; Schwob et al., 2017). Neuronal regeneration is a distinctive feature of the OE: few areas of the nervous system are known to have regenerative ability.

To detect odorants, ORNs express the largest family of G protein–coupled receptors (GPCRs) so far discovered: the odorant receptors (ORs). Interestingly, ORs have increasingly been described in several other organs (Flegel et al., 2013; Maßberg and Hatt, 2018). In the OE, these receptor proteins can bind and be activated by different odorant molecules with different chemophysical features and trigger a signal transduction cascade that ultimately leads to electrical signals (action potentials) traveling to the olfactory bulb (OB; reviewed in Menini et al., 2004; Reisert and Zhao, 2011; Pifferi et al., 2012). Thus, as an island of neurons in the sea of the external world, the OE is an outpost that can potentially inform our knowledge about the brain’s physiological and pathological status.

In neurodegenerative diseases such as Parkinson’s disease (PD) and Alzheimer’s disease (AD), olfactory dysfunction appears relatively early compared to other symptoms (Doty, 2009, 2012; Hummel et al., 2017). In idiopathic PD, olfactory loss can occur several years prior to the onset of the motor symptoms (Doty, 2012). Indeed, olfactory dysfunction has been designated as one of the diagnostic criteria for PD since 2006 (Suchowersky et al., 2006).

Our understanding of the olfactory pathophysiology for AD is less clear than that for PD, and it is still unclear which neural substrates are responsible for AD (Doty, 2017). In this review, we summarize studies on the involvement of the olfactory system in AD, with a focus on events during the early stages of olfactory processing, in the OE and OB. We first outline the development and function of the OE and OB, followed by an overview of the hypotheses of AD development and pathology, before discussing the interrelation of AD with the olfactory system. Several studies (reviewed in Murphy, 2019) support the notion that olfactory dysfunction develops before cognitive decline and prior to signs of dementia in patients with AD, in those with a genetic risk of AD, and with amnesic mild cognitive impairment. Thus, changes in the early stages of olfaction have the potential to be an inexpensive and non-invasive diagnostic tool and biomarker. Currently, the use of olfactory function as the sole predictor of the AD is limited (Velayudhan, 2015).

## The Olfactory System

### The Olfactory Epithelium

**FIGURE 1:**
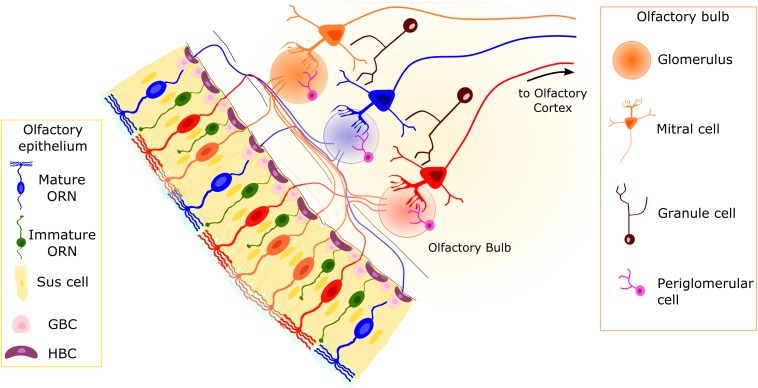
Organization of the olfactory system. The pseudostratified olfactory epithelium (OE) consists of sustentacular (Sus) cells, olfactory receptor neurons (ORNs), and basal cells. The Sus cells have a columnar cell body, with an apical membrane with microvilli. Two main populations of neurons are present: mature and immature ORNs. Both ORN types are bipolar neurons with dendritic processes projecting toward the apical surface of the epithelium. In mature ORNs, several cilia protrude from the dendritic knob. Horizontal basal cells (HBCs) and globose basal cells (GBCs) constitute the stem cells of the OE. They are able to replace the different cell types on a daily basis or in case of injury. Axons from ORNs expressing the same odorant receptor type (represented by ORNs of the same color) converge in the olfactory bulb (OB) in round structures dense with synapses, called glomeruli. The second-order neurons (mitral/tufted cells) have a single dendrite projecting to a single glomerulus and lateral dendrites that contact dendrites of granule cells.

Two main neuronal cell populations are present in the OE: immature and mature ORNs. Olfactory receptor neurons are bipolar neurons with a cell body located in the middle part of the OE and a dendrite that extends toward the nasal cavity, ending with a knob bearing several cilia. At the other end of the cell body, an axon exits the OE from the basal lamina and reaches the OB in the brain (Figure 1). Mature ORNs can be identified because of their higher expression of olfactory marker protein, a cytosolic protein involved in the odorant response (Buiakova et al., 1996; Ivic et al., 2000; Reisert et al., 2007; Lee et al., 2011; Dibattista and Reisert, 2016) and widely used as a molecular marker or driver for the expression of genes in mature neurons. Mature ORNs express all the proteins to elicit the odorant response, including the ORs (reviewed in Kleene, 2008), which are GPCRs expressed in ORN cilia. Odorant receptors represent by far the most numerous class of genes in vertebrate genomes, representing on average 3–5% of all genes across species, and were first elegantly cloned from the rat OE in 1991 by Linda Buck and Richard Axel (Axel and Buck, 1991). Different ORs bind different odorant molecules with many different chemical features, and they can be either broadly tuned or narrowly selective.

Odorant signal transduction takes place in the olfactory cilia. It starts with the binding of an odorant molecule to an OR, which triggers a series of molecular events mediated by the second messenger cyclic adenosine monophosphate (cAMP), generated by adenylyl cyclase type 3 (AC3). The odorant-induced increase in ciliary cAMP generates a transduction current carried by Na^+^ and Ca^2+^ via entry through the cyclic nucleotide-gated channels first, followed by Cl^–^ exit through Ca^2+^-activated Cl^–^ channels (TMEM16B or anoctamin-2; reviewed in Pifferi et al., 2006; Kleene, 2008; Pifferi et al., 2012; Dibattista et al., 2017). The ensuing depolarization generates action potentials that are then sent to the OB.

The OE contains cell types other than the functional units of the olfactory system, the ORNs (Figure 1; Andres, 1966; Graziadei and Graziadei, 1979; Sokpor et al., 2018):

1) Microvillar sustentacular or supporting cells (Nomura et al., 2004; Sokpor et al., 2018);
2) Microvillar cells, a relatively small heterogeneous population in terms of phenotype and function (Morrison and Costanzo, 1989; Moran et al., 1992; Asan and Drenckhahn, 2005; Montani et al., 2006; Hegg et al., 2009; Jia et al., 2013; Kusumakshi et al., 2015); and
3) At least two populations of basal cells that constitute the stem cell niche of the OE: horizontal or dark basal cells (HBCs) and globose basal cells (GBCs; Figure 1; Graziadei and Graziadei, 1979; Huard and Schwob, 1995; Schwob et al., 2017).

Microvillar sustentacular cells are goblet-shaped cells with a basal end foot that contacts the OE basal lamina and microvilli located at their apical side. They are thought to play a range of roles, from detoxification of the OE to odorant transformation (Rendic and Di Carlo, 1997; Thiebaud et al., 2013). They produce neurotrophic and neuromodulator molecules such as endocannabinoids, insulin, and ATP (Czesnik et al., 2006; Breunig et al., 2010; Hayoz et al., 2012; Henriques et al., 2019). Interestingly, several studies have shown that ATP is involved in neuroprotection and neuroproliferation (Hassenklöver et al., 2009; Jia and Hegg, 2010; Jia et al., 2013).

Both types of basal cell populations are multipotent, but HBCs are considered reserve stem cells, whereas GBCs are responsible for continuous replacement of ORNs and other cell types in the OE. Horizontal basal cells are usually dormant in the OE, but severe OE lesions or toxin-mediated injury wakes up this stem cell population, which gives rise to a plethora of cell types, including ORNs and sustentacular cells (reviewed in Schwob et al., 2017). Globose basal cells are round-shaped cells with a relatively small cytoplasm, located in clusters separated by gaps. Among the heterogeneous population of GBCs are those that will give rise to the mature ORNs (Graziadei and Graziadei, 1979; Schwob et al., 2017).

### The Olfactory Bulb

The OB is the first relay station of the olfactory system in the brain (Figure 1). Olfactory receptor neurons expressing the same OR type convey their axon to neuropil-like structures in the OB called glomeruli. Thus, the OB is topographically organized, with each glomerulus representing a single type of OR. Glomeruli are surrounded by periglomerular cells (PGCs) and are rather uniform in size (approximately 50-μm diameter in mice). In the glomeruli, ORN axons connect to the apical dendrite of mitral/tufted cells, whose cell bodies are located in the external and internal plexiform layers, where GABAergic granule cells (GCs) also reside (Figure 1). Olfactory receptor neurons also make synapses onto several populations of PGCs.

The mechanisms by which ORN axons reach the correct glomerulus in the OB and form the glomerular structure are not fully understood. During early development and map formation, axons project to stereotypical areas along the dorsoventral and anteroposterior axes in the OB. Molecules such as neuropilins, ephrin As, and semaphorins act as guidance cues expressed by ORN axons (Cutforth et al., 2003; Imai et al., 2006; Takeuchi et al., 2010). The mechanism involved in the map formation along the anteroposterior and dorsoventral axes seems to be mainly cAMP dependent at first. Recently, Wu et al. (2018) showed that a population of immature ORNs can act as “navigators,” mediating the initial coalescing of axons into protoglomeruli in the OB.

In addition, other guidance molecules, whose expression seems OR dependent, play important roles in allowing axons expressing the same OR to target the same glomerulus (Kaneko-Goto et al., 2008; Nakashima et al., 2013; Takeuchi and Sakano, 2014). Ligand-dependent activity seems to regulate the expression of guidance cues that drive axon coalescence and glomerular refinement (Lodovichi and Belluscio, 2012; Takeuchi and Sakano, 2014; Yu and Wu, 2017). Odorant receptors may also mediate axon targeting directly by binding small peptides (Zamparo et al., 2019). The formation of the correct glomerular map is considered important for odor coding; alteration in the map could lead to deficits in odor coding and, ultimately, in olfactory-driven behaviors.

## Pathophysiological Mechanisms of Alzheimer’s Disease

Alzheimer’s disease is a devastating neurodegenerative disorder that affects 47 million people globally (Dementia Statistics Alzheimer’s Disease International, 2017). As yet no cure for AD exists, and as AD progresses, memory loss and cognitive disorder present debilitating problems for patients and increased burden for caregivers.

Alzheimer’s disease is characterized by a long, insidious progression, with a preclinical phase that can last from 5 to 15 years before the onset of disease symptoms, which then worsen in the symptomatic phase that lasts up to 10 years (Long and Holtzman, 2019). It is important to understand how the disease progresses and whether it is possible to identify any biomarker in the very early stage of the disease prior to the manifestation of cognitive symptoms.

### Amyloid and Tau Cascade

Alzheimer’s disease has two specific neuropathological markers: first, extracellular deposition of amyloid-β (Aβ) protein as diffuse and neuritic plaques and, second, dystrophic neurites and intraneuronal neurofibrillary tangles (NFTs) composed of aggregated hyperphosphorylated tau protein (Jack et al., 2018). In the earliest working hypothesis of AD pathogenesis, known as the amyloid cascade hypothesis (Hardy and Higgins, 1992), the initial step of the disease is the deposition of Aβ, followed by subsequent tau deposition, neurodegeneration, and ultimately cognitive decline. This amyloid hypothesis has recently been extended to include inflammatory mechanisms (described in *ApoE and the Inflammatory Hypothesis*).

**FIGURE 2:**
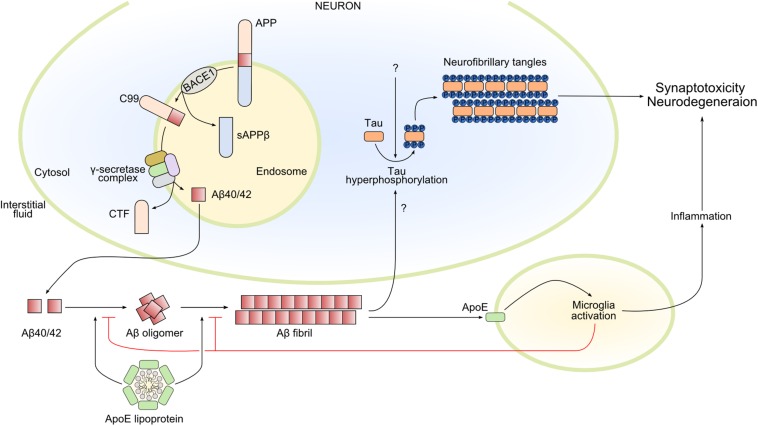
Main pathways involved in Alzheimer’s disease pathophysiology. Amyloid-β 40/42 (Aβ_40__/__42_) is produced by the sequential cleavage of amyloid precursor protein (APP) by BACE1 (β-secretase 1) and γ-secretase complex in the endosome. After secretion into the interstitial fluid, Aβ_40__/__42_ forms oligomers and Aβ fibrils that contribute in different ways to Alzheimer’s disease pathology. In particular, Aβ fibrils activate the hyperphosphorylation of tau, promoting its oligomerization and the formation of intracellular neurofibrillary tangles, leading to synaptotoxicity and neurodegeneration. Amyloid-β fibrils also activate microglia by apolipoprotein E (ApoE)–dependent mechanisms. Microglia activation can have both beneficial and damaging effects: microglia reduce and delay Aβ fibril formation, but the prolonged release of inflammatory mediators could lead to neurodegeneration. Finally, ApoE lipoprotein also modulates Aβ oligomerization and Aβ fibrils. CTF, C-terminal fragment; sAPPβ, soluble APP β; C99, the 99-amino acid C-terminal domain of APP.

The initial deposition of Aβ begins with a series of secretase enzymes (MMP24, ADAM10, BACE1, PSEN1, and PSEN2) that cleave the membrane protein APP (amyloid precursor protein), in sequence and at different sites; some of the resulting products are Aβ_40_ and Aβ_42_ (Figure 2). The proteolytic function of BACE1 and γ-secretase seems to be mainly in the intracellular endosomes; the combined action of the complexes of these two secretases forms Aβ from APP. The polypeptides Aβ_40_ and Aβ_42_ have a tendency to aggregate to form higher-order oligomers with a wide molecular weight range, which eventually leads to the formation of fibrils (Sakono and Zako, 2010). The importance of Aβ aggregation in triggering the AD cascade is backed by genetic data, although Aβ seems to be necessary but not sufficient (Long and Holtzman, 2019).

None of the Aβ peptides is intrinsically toxic; rather, a change in their ratio, favoring the more insoluble form, is linked to AD. For example, Aβ_40_ formation is altered when PSEN1 is mutated, thus favoring an increased Aβ_42_/Aβ_40_ ratio (Kametani and Hasegawa, 2018). Minor changes in the ratio can drive the seeding of plaques and, via an independent mechanism, neurotoxicity. In other words, a mixture of various oligomers and aggregates (an “Aβ cocktail”) and not one specific toxic oligomer exists to interact with cellular membranes and proteins in non-specific ways (Benilova et al., 2012).

A driver of neurodegeneration in AD is the tau protein in its aggregated and hyperphosphorylated forms (Figure 2). There might be a link between Aβ deposition and tau protein homeostasis, such that elevated Aβ formation is sufficient to drive tau pathology. Tau protein is encoded by the *MAPT* (microtubule-associated protein tau) gene and is primarily expressed by neurons in the brain. It is alternatively spliced at the N-terminal domain (N) and microtubule-binding repeat domain (R), which yields six distinct isoforms: 0N3R, 0N4R, 1N3R, 1N4R, 2N3R, and 2N4R. How tau functions in the central nervous system is not fully elucidated. It seems to be involved in microtubule assembly, stabilization of neuronal axons, and regulation of microtubule-associated transport (Weingarten et al., 1975; Dixit et al., 2008).

In several cases, because of the many different phosphorylation sites of the tau protein, unusual phosphorylation causes a decrease in binding affinity for microtubules (Biernat et al., 1993; Mandelkow et al., 2007). Tau then cannot bind correctly to the microtubular structure, resulting in an increase of the cytosolic pool that can promote aggregation and fibrillization. Relocation of hyperphosphorylated tau from the axonal to other cellular compartments, like dendrites and the soma, can then lead to decline of synaptic function (Braak and Tredici, 2016). It seems that tau is mainly degraded in the cell body and continues to accumulate in the axon, which lacks degrading enzymes. This scenario makes it possible for misfolded hyperphosphorylated tau protein to infiltrate into terminal branches of the axon and into presynaptic terminals. Once there, tau can be transported across the synaptic cleft, causing connected neurons, via their synapses, to become susceptible to AD pathology. These last events, if proven, could support considering AD a “prion-like” disease, where misfolded proteins spread from one cell to another and trigger pathogenic mechanisms in otherwise healthy neurons (Kametani and Hasegawa, 2018). Numerous studies have shown prion-like self-propagation of aggregated human tau fibrils, both synthetically prepared and those derived from human AD brain (Condello and Stöehr, 2018; Aoyagi et al., 2019).

### Apolipoprotein E and the Inflammatory Hypothesis

In AD pathology, the progression of AD involves not only neurons but also astrocytes and microglia. These cell types in the brain express apolipoprotein E (ApoE), a protein encoded by a gene that is the strongest genetic risk factor for AD (Corder et al., 1993; Strittmatter et al., 1993). Several studies in the last two decades, including clinical, pathological, epidemiological genome-wide association, and whole-genome sequencing studies, have confirmed the strength of the association between ApoE and AD (Shi et al., 2017; Kunkle et al., 2019). Apolipoprotein E predominantly functions as a lipid-binding protein in lipoprotein particles, and it participates in transport and delivery of lipids to target sites (Figure 2). In AD, it seems to be present in Aβ plaques (Namba et al., 1991). Interestingly, the *APOE* gene has three common alleles encoding three protein isoforms: ApoE2, ApoE3, and ApoE4. During the preclinical phase of AD, carriers of the ApoE4 allele increase cerebral amyloid deposition at an earlier age and do so faster than non-carriers (Grimmer et al., 2010; Morris et al., 2010; Risacher et al., 2013; Bussy et al., 2019). Apolipoprotein E seems to exert its effects on neurodegeneration independently from Aβ plaques by modulating tau pathology. Apolipoprotein E4 might make neurons more sensitive to neurodegeneration in the tauopathy mouse model (tauP301S), while its absence may be protective (Shi et al., 2017). Moreover, lack of ApoE greatly reduced activation of microglia and astrocytes in this model (Shi et al., 2017). Whether ApoE could be defined as a possible missing link between amyloid deposition, tau pathology, and neuroinflammation is not known.

Recently, the amyloid hypothesis has been extended to what can be called the “inflammatory hypothesis,” as an increasing number of inflammatory mechanisms have been implicated in AD pathogenesis (Kinney et al., 2018; Long and Holtzman, 2019). Reactive astrogliosis and microgliosis are important pathological features of AD. Microglia play apparently paradoxical roles: protective during amyloid deposition but damaging during tau accumulation. Microglia help clear Aβ during the early stage of AD, but during progression their continued release of proinflammatory cytokines and associated neurotoxins is thought to cause neurodegeneration, which in turn activates more microglia, triggering a vicious cycle. Interestingly, removing microglia during the period of plaque deposition seems to have a protective effect (Sosna et al., 2018), thus confirming that activated microglia could be involved in some of the changes in the brains of AD patients. Activated microglia also augment the negative effects of tau accumulation (Lee et al., 2013).

### The Infectious Hypothesis

The so-called infectious hypothesis, first proposed in 1991 (Jamieson et al., 1991), has recently received renewed attention. It was first reported as a correlation of herpesvirus in the brains of AD patients and in amyloid plaques. The infectious hypothesis gained popularity recently when specific viral DNA, belonging to viruses of the family Herpesviridae, was found in three independent cohorts of AD patients, and its presence was then linked via multiscale network analysis to aging and AD development (Readhead et al., 2018). The proposed pathophysiology supports the idea that abnormal processing of APP to Aβ is aided by the presence of several types of herpes simplex virus (HSV-1, HSV-6, and HSV-7) in the brain, leading to toxic Aβ aggregation and the hyperphosphorylation of tau (Itzhaki, 2016). Several other microorganisms (ranging from viruses to bacteria) have been linked to AD or, rather, to the hypothetical antimicrobial function of Aβ, which seems to have characteristics similar to antimicrobial peptides (Soscia et al., 2010). In this view, Aβ fibrillization is a defensive mechanism that triggers the amyloid cascade (Moir et al., 2018).

The infectious hypothesis could also partially explain and/or integrate with the role of neuroinflammation as a protective response toward microbial challenge, thus releasing the antimicrobial peptide Aβ, rather than as a consequence of Aβ deposition, as viewed by the amyloid cascade hypothesis.

## Olfaction and Alzheimer’s Disease

Olfactory impairments, centrally and peripherally, often appear in the early phase of AD, before cognitive impairment is apparent in patients (Murphy, 2019). In these cases, the olfactory system shows the classical signs of AD: intracellular NFTs and Aβ plaques. Amyloid-β peptides, generated from APP, aggregate into extracellular plaques, whereas NFTs result from intracellular accumulation of hyperphosphorylated tau proteins (Cai et al., 2012; Attems et al., 2014; Franks et al., 2015). In postmortem tissues from AD patients, both Aβ plaques and NFTs are found in both OE and OB (Talamo et al., 1989). Amyloid-β and tau expression can also be found in cells swabbed from the nasal cavity of healthy human subjects (Brozzetti et al., 2020).

### The Olfactory Epithelium in Alzheimer’s Disease

Several studies in animal models have investigated how the different pathways involved in AD may affect the function of the OE, possibly contributing to olfactory impairment as observed in AD patients.

In a transgenic mouse model that selectively overexpresses the mutated humanized APP (hAPP, with the so-called Swedish and Indiana mutations that facilitate Aβ generation) in either mature or immature ORNs, mature ORNs are more susceptible to stress induced by APP accumulation (Cheng et al., 2011). Intracellular hAPP accumulation seems to induce neuronal loss, indicating that mechanisms involved in neuronal apoptosis might be cell autonomous (Cheng et al., 2011, 2016). The researchers did not observe extracellular plaques when apoptosis of ORNs was evident in the OE and showed that only ORNs expressing hAPP were sensitive to its accumulation. Those not expressing the protein were spared from apoptosis. In wild-type mice, nasal injection of an adenovirus expressing either Bri-Aβ_40_ or Bri-Aβ_42_ fusion proteins, which are cleaved to secrete the human Aβ_40_ or Aβ_42_ peptide, respectively, did not increase apoptotic cells in the OE but, rather, disrupted correct axonal targeting to the OB, without any clear visible sign of plaque deposition (Cao et al., 2012b).

On the other hand, cell death in the OE seems not to be exclusively cell autonomous. The Tg2576 mouse model expressing hAPP with naturally occurring Swedish mutations (K670N, M671L, hAPPsw), which increases Aβ production by γ-secretase, shows an unusually high accumulation of Aβ_56_, a neurotoxic form of Aβ (Kim et al., 2018). Amyloid-β_56_ is a dodecamer of Aβ that, when accumulated in the cortex, seems to be related to cognitive impairments (Benilova et al., 2012). Interestingly, Aβ_56_ accumulates in the OE before mice show cognitive deficits (Kim et al., 2018). The researchers speculated that the increase of the neurotoxic Aβ_56_ could be explained by an upregulation of PSEN1 and PSEN2 only in the OE (Kim et al., 2018). Thus, it is possible that APP-processing machinery in the OE differs from that in other regions of the brain.

A later study by the same group showed that the rate of apoptosis in the OE in Tg2576 mice seems to be region specific (Yoo et al., 2017), indicating that it is possible to dissect out the early signs of AD in defined zones of the OE. The rate of apoptosis also correlated with Aβ accumulation, which was higher in regions with more apoptotic ORNs. This suggests that susceptibility to Aβ accumulation even within the OE might be differently regulated. It is interesting to note that ORs are expressed in specific zones (Ressler et al., 1993) and that the dorsal OE region displays slowed ORN turnover (Vedin et al., 2009), suggesting that the region-specific effects of AD might be another sign of heterogeneity of the OE, or potentially linked to mechanisms that drive the aforementioned OE variations. However, transgenic mice overexpressing APP, with a single or multiple amyloidogenic mutant human genes, have mutations not present in the human sporadic form of AD, and the temporal progression of the disease in mice does not correlate well with AD in humans (Masurkar and Devanand, 2014). Thus, these results should be interpreted with caution.

In AD patients, OE supporting cells seem to undergo the same modification and decrease in number as ORNs (Brouillard et al., 1994). Moreover, it has been shown in mice that, after adenoviral infections with either Aβ_40_ or Aβ_42_, the sustentacular cells clearly contain these protein fragments, perhaps indicating their potential role in the uptake and phagocytosis of Aβ (Cao et al., 2012b), similar to the role played in the brain by microglia, astrocytes, and macrophages (Ries and Sastre, 2016).

An aspect that seems peculiar to the olfactory system is that Aβ burden correlates with olfactory impairment (Wesson et al., 2010); Aβ plaque accumulation, in contrast, does not correlate with the progression of cognitive impairment (Sakono and Zako, 2010; Long and Holtzman, 2019; Murphy, 2019), although NFT accumulation does correlate with behavior impairments (Murphy, 2019). Because no tau mutations have been found in sporadic AD, mouse models for tau are mainly considered models for dementia (Jankowsky and Zheng, 2017), and tau expression might not recapitulate the evolution of NFTs spreading between synaptically connected regions (Gendron and Petrucelli, 2009; Jankowsky and Zheng, 2017). A transgenic mouse overexpressing tau protein showed behavioral olfactory deficits and intense tau immunoreactivity in the OB (Macknin et al., 2004).

In postmortem tissues of patients with and without AD, multiple tau protein domains in NFTs have been described in the OE, in what look like ORN axons (Lee et al., 2011). More recent studies found higher levels of hyperphosphorylated tau in the OE of AD subjects than in those suffering from other neurodegenerative diseases, and its expression with a morphological appearance typical of NFTs is localized to ORN cell bodies (Arnold et al., 2010). Deficits in olfactory behavior were demonstrated in the 3xTg-AD mouse model for human AD, which expresses mutations in PSEN1, APP with the Swedish mutations, and mutated tau, leading to development of Aβ and tau pathologic lesions in a sequential manner (Oddo et al., 2003; Cassano et al., 2011). However, it is not clear whether Aβ or tau accumulation is responsible for the altered behavior or whether they accumulate in the OE (Cassano et al., 2011).

An ApoE genotype seems to manifest itself uniquely and early in human ApoE4 carriers with the appearance of olfactory dysfunction (Price et al., 1991; Bacon et al., 1998; Mesholam et al., 1998; Murphy et al., 1998; Graves et al., 1999; Gilbert and Murphy, 2004; Peng et al., 2017), impaired odor identification (Murphy et al., 1998; Olofsson et al., 2016), and altered olfactory-related evoked potentials (Kowalewski and Murphy, 2012; Morgan and Murphy, 2012). This occurs before other forms of cognitive impairment, for example, impairment associated with Aβ and tau pathology. Previous studies have shown ApoE expression in the sustentacular cells (Nathan et al., 2007). As a consequence of ApoE deletion in mice, OE recovery from an injury is slowed down (Nathan et al., 2010). This suggests involvement of sustentacular cell ApoE in regulating ORN maturation and survival under physiologically stressful conditions. Apolipoprotein E from sustentacular cells and ensheathing glia could play important roles in recycling membrane components liberated from senescing ORNs to support cell division and differentiation of basal cells and axonal outgrowth of maturing ORNs. Apolipoprotein E in sustentacular cells may facilitate the traditional supporting role attributed to these cells in OE and to glial cells in the brain (Getchell et al., 1984; Ramón-Cueto and Avila, 1998; Barnett, 2004).

### Odorant Receptors in Alzheimer’s Disease

A growing number of reports describe ORs expressed in tissues other than the OE (Maßberg and Hatt, 2018). Those ORs have traditionally been called ectopic ORs, or “ecto-nomotopic,” to highlight the possibility that the normal function of ORs might not be limited to olfactory perception (Di Pizio et al., 2019). Often, ectopic ORs are expressed with other elements of the canonical olfactory signal transduction machinery, such as olfactory G protein G_olf_, AC3, and receptor-transporting proteins. Since the very first description of an OR outside the nose [in testis; (Spehr et al., 2003)], several reports have shown OR expression in rodent and human non-chemosensory tissues and organs, such as lung (Kalbe et al., 2017), skin (Gelis et al., 2016), kidney (Pluznick et al., 2013; Shepard and Pluznick, 2016), pancreas (Munakata et al., 2018), brain (Otaki et al., 2004; Flegel et al., 2013; Grison et al., 2014), and a variety of other locations (reviewed in Maßberg and Hatt, 2018). However, few well-described physiological functions have been identified for ectopic ORs (Pluznick et al., 2013; Shepard and Pluznick, 2016; Bellono et al., 2017). Their possible implications in pathophysiological processes are also elusive. In postmortem tissues of subjects without signs of neurodegenerative diseases, several ORs have been found to be expressed in frontal cortex, entorhinal cortex, and cerebellum. For example, human OR51E1 is one of the few ORs whose expression outside the nose seems higher than in the OE (Flegel et al., 2013), and along with other ORs, it seems to be expressed also in the brain (see Ansoleaga et al., 2013). Some ORs were found to be differentially regulated at different stages of AD and in the different brain regions (Ansoleaga et al., 2013). In addition, a progressive increase in Olfr110 mRNA expression and decreased expression of AC3 in the brain were found at different ages in the hAPPsw/PSEN1 mouse, another mouse model of AD (Ansoleaga et al., 2013). Interestingly, during AD progression, a stage-dependent regulation of ORs seems to exist in the OB. Recent data of postmortem human OBs showed a transcriptomic fingerprint related to the different stages of AD progression: among the involved genes were OR2T2 and OR5M1, downregulated at intermediate stages, and OR2T8 and OR6J1 genes upregulated in advanced stages (Lachén-Montes et al., 2017b). Worth noticing is also the presence of ORs in the OB in a still unknown cell type. Transcriptomic analysis may be worth extending to the OE where data about changes in OR expression profile during AD progression are not available yet.

Together with Olfr110, other ORs have been described in the brain of wild-type mice: Olfr110/111 and Olfr544 proteins are mainly expressed in cortical and hippocampal neurons and at lower levels by astrocytes, microglia, oligodendrocytes, and endothelial cells (Gaudel et al., 2019). The expression of these ORs was evaluated in the 5xFAD transgenic mouse model for AD. These mice exhibited upregulated expression of Olfr110/111 in cortex and hippocampus, and Olfr544 was overexpressed only at 9 months of age in cortex (Gaudel et al., 2019).

A different set of experiments with a different animal model (knock-in for the different human ApoE isoforms), surprisingly, showed that Olfr110 is regulated differently by the different ApoE forms during induced microglia activation (Shi et al., 2017). Cultured microglia treated with lipopolysaccharide showed upregulation of proinflammatory genes, including *Olfr110* (Shi et al., 2017), which may suggest a potential proinflammatory chemosensing role for this OR, which is poorly expressed in the nose and is enriched in other brain areas (Gaudel et al., 2019). The possibility that ORs may function outside the OE is intriguing because it opens an array of new questions about physiological roles of ORs and their potential roles in AD.

### Electrophysiological Responses of the OE in Alzheimer’s Disease

Few studies have addressed the electrophysiological properties of ORNs or of the entire epithelium in AD. The OE in humans is accessible for electrophysiological recordings, and its response to odorants can be measured (Lapid et al., 2011; Lapid and Hummel, 2013). Human ORNs are also accessible for biopsy and subsequent functional investigation (Rawson et al., 1998).

ApoE-knockout (KO) mice display a deficit in odorant responses recorded from the OE surface via electro-olfactograms (Zhang et al., 2018), which represent the summed generator potential recorded from multiple ORNs in the OE that are activated by an odorant. Hence, electro-olfactograms depend on the number of responsive ORNs in the OE and could also reflect the change of function of transduction proteins (Pinato et al., 2008; Song et al., 2008; Cygnar and Zhao, 2009; Cygnar et al., 2010). Apolipoprotein E–KO mice showed both a deficit in the amplitude of odorant responses and a lack of adaptation at ages 3–5 months (Zhang et al., 2018). To our knowledge, no further electrophysiological data are available of mouse models for AD.

It would be interesting to know more about what changes occur when ORNs begin to accumulate proteins that trigger apoptotic events in AD. Changes in intrinsic neural activity, including hyperactive and hypoactive neural systems, in mouse models of AD have been reported for a number of different types of neurons, including early-life hyperactivity in the OB that may result from Aβ and other APP metabolites (Wesson et al., 2011; Rey et al., 2018). Some ORNs are characterized by higher spontaneous activity, determined by the OR type they express (Reisert, 2010; Connelly et al., 2013). This spontaneous activity dictates the physiology of these ORNs (Dibattista and Reisert, 2016) and their targeting to the OB (Imai et al., 2006; Nakashima et al., 2013; Takeuchi and Sakano, 2014). The role of BACE1 (β-site amyloid precursor protein cleaving enzyme 1) in glomerular targeting is zone specific (Yoo et al., 2017) or OR dependent (Rajapaksha et al., 2011; Cao et al., 2012a). It is tempting to speculate that the different phenotypes may be dependent on the spontaneous activity of the ORs and the ORNs.

Mitochondrial dysfunction has received increased attention as one of the main mechanisms that can cause sporadic late-onset AD (Cenini and Voos, 2019). Mitochondria in the dendritic knob contribute directly to Ca^2+^ homeostasis and are responsible for Ca^2+^ clearance during odorant stimulation. It is noteworthy that pharmacologically altering the inner mitochondrial membrane potential leads to a shift of the dynamic range of the ORN stimulus–response function and detrimentally affects odorant responses (Fluegge et al., 2012). Thus, early events during AD that may involve mitochondria could alter olfactory transduction and could be detected by measuring ORN activity (Fluegge et al., 2012).

### Olfactory System Neuroregeneration in Alzheimer’s Disease

As the peripheral olfactory system regenerates throughout life, this imposes additional challenges during the progression of AD both for newly born ORNs and the integration of their axons in the bulbar circuitry.

The OE basal cells are responsible for the replacement of all OE cell types throughout the life span (Graziadei and Graziadei, 1979; Schwob et al., 2017). As basal cells differentiate into mature ORNs, they pass through a stage as immature ORNs, characterized by the expression of GAP43, which has been extensively used as a marker for this cell population. In a transgenic mouse model, overexpressing mutated hAPP in either immature or mature ORNs was sufficient to induce neuronal cell death (Cheng et al., 2011). One difference, though, was that the rate of immature cell production was increased. Interestingly, this animal model allowed temporal control of hAPP expression, and after a period of hAPP production, the researchers shut off its expression and found that immature neurons were less susceptible than mature ORNs to apoptosis. Also, immature ORNs recovered from neurodegeneration faster than mature ORNs (Cheng et al., 2011). This study is the only reported exploitation of OE stem cells in AD research. Note that in both mice and humans the OE tends to lose its neuroregenerative ability with age (Child et al., 2018): GBCs somehow run out, HBCs alone are no longer sufficient, and the OE becomes non-neurogenic (Schwob et al., 2017).

Neuroregeneration also occurs in the OB. The synapses of ORNs are subject to renewal as, for example, PGCs are continuously integrated into the glomerular circuit even in adult mice (Lepousez et al., 2013), although PGCs represent only ∼5% of the total number of newborn neurons in the OB (Batista-Brito et al., 2008; Lepousez et al., 2013). Different specialized precursor populations in the subventricular zone (SVZ) are thought to generate the different PGCs (Merkle et al., 2007). Following sensory deprivation, survival of new PGC declines (Mandairon et al., 2006), whereas survival of adult-generated PGCs can be increased by olfactory enrichment and learning (Alonso et al., 2006; Lepousez et al., 2013).

Granule cells are GABAergic, axonless interneurons, releasing GABA across a reciprocal dendrodendritic synapse with mitral and tufted cells. During adulthood, newborn GCs in many mammalian species migrate from the SVZ of the anterior forebrain to the OB where they integrate into the OB circuitry, where the maturation of the immature GCs is uniquely influenced by sensory and top-down activity (reviewed in Lepousez et al., 2013).

In the hAPPsw/PSEN1 mouse model, where both hAPPsw and mutated PSEN1 are present, Aβ deposition is restricted to the GC layer (Saiz-Sanchez et al., 2013). Granule cells are a subpopulation of somatostatin-expressing neurons that decrease with age, or abruptly in hAPPsw/PSEN1 mice (De la Rosa-Prieto et al., 2016). Transgenic mice that express single mutant variants of APP or PSEN1 showed several different neurogenic phenotypes (Lazarov et al., 2010) during the progression of AD symptoms. Neurogenesis either increased or decreased in response to neurodegenerative processes (Chen et al., 2008; Saiz-Sanchez et al., 2013). Different results regarding the rate of neurogenesis may depend on amyloid plaque deposition in the SVZ, which appears to be toxic to progenitor cells. This suggests that survival of progenitor cells in the SVZ is reduced in AD, and the net result is decreased SVZ proliferation (Curtis et al., 2007).

### The Olfactory Bulb in Alzheimer’s Disease

In adult rats, APP protein is highly expressed in the OB, olfactory nerve layer, external plexiform layer, and glomerular layer, and damage to the OE increases expression of APP in the OB (Struble et al., 1998). In humans, Aβ deposition occurs in the OB prior to the appearance of cognitive symptoms (Attems et al., 2014). In the Tg2576 mouse model, which overexpresses hAPPsw, non-fibrillary Aβ deposition has been observed earlier in the OB than in other brain areas (Wesson et al., 2010). Furthermore, axonal targeting of ORN axons to glomeruli in the OB is disrupted in mice bearing the hAPPsw mutation (Cao et al., 2012b).

The effects of overexpressing hAPPsw have been investigated in an OR-dependent manner by crossing mice that express hAPPsw only in mature ORNs with mouse lines that express the GFP or LacZ reporter in ORNs expressing the *P2*, *MOR28*, and *M71* OR genes. These mice showed that axonal projections terminate within multiple glomeruli per half bulb (Cao et al., 2012b) instead of in a single glomerulus. The ORN-specific restricted expression of hAPPsw and the bulbar mistargeting allowed the researchers to determine how these connectivity deficits altered odorant-driven behaviors. They found that mice with the amylogenic mutations needed increased time to find a hidden food source and spent more time close to where a repulsive fox-urine–derived compound had been deposited, suggesting that these mice had reduced olfactory abilities.

In addition, the researchers tested for odorant responses in the OB of hAPPsw mice by monitoring tyrosine hydroxylase (TH) expression as a proxy for ORN-driven input activity, using immunofluorescence. They showed that periglomerular dopaminergic neurons had reduced TH expression. They also tested for reduced expression of the immediate–early gene *Arc* in periglomerular and tufted neurons (Cao et al., 2012b). Because periglomerular and tufted neurons in the OB do not express hAPPsw themselves, these reduced levels of TH and *Arc* are thought to be caused by reduced input from ORNs (Cao et al., 2012b). It is noteworthy that the more neurotoxic Aβ_56_ form seems to be present in the early phase of AD in adult Tg2576 mouse ORNs (see *The Olfactory Epithelium in Alzheimer’s Disease* and Yoo et al., 2017), and the decreased ORN activity may be due to its accumulation.

An interesting aspect of the work by Cao et al. (2012b) is the use of mice with ORN-specific overexpression of either hAPPwt (a non–Aβ-forming form of human APP) or hAPPmv (which does not allow BACE1 to cleave APP), which did not reveal any connectivity defects. This reinforces the idea that expression of hAPPsw produces Aβ as a product of APP cleavage by BACE1 in mice *in vivo* and alters the neuronal circuitry of the peripheral olfactory system in the absence of plaques. The mistargeting phenotype is observed without any plaques in both young and adult mice. Indeed, the same phenotype can be found in adult wild-type mice by virally expressing Aβ_40_ or Aβ_42_ in the OE. Together, these data are consistent with soluble Aβ triggering axonal dysfunction and mistargeting in the absence of amyloid plaques *in vivo* (Cao et al., 2012b).

The appearance of a specific AD phenotype in the absence of plaques has also been shown recently in the OB by proteomics and analysis of mice expressing hAPPsw protein (Lachén-Montes et al., 2019). Using these methods, the researchers were able to differentiate variations in protein and transcript levels that were peculiar in the various stages of AD progression (i.e., early vs late stage of AD) (Lachén-Montes et al., 2019). When APP is overexpressed, AD stage-related regulatory effects on kinase dynamics (SEK1/SAPK, PKA) could be detected in the OB. Interestingly, these same kinases are differentially affected in OB samples from patients at different stages of AD progression (Lachén-Montes et al., 2017a, 2019).

BACE1 is necessary to generate Aβ peptide, but in the olfactory system, it seems to play a role in glomerular targeting in the OB. The precise targeting of ORN axons is disturbed in BACE1-KO mice (Rajapaksha et al., 2011; Cao et al., 2012a). BACE1 protein localizes mainly to the proximal segment and the terminus of ORN axons, and the expression of BACE1 correlates inversely with odor-evoked neural activity. Crossing BACE1-KO mice with mice whose ORs have been tagged with a GFP or LacZ reporter (Rajapaksha et al., 2011; Cao et al., 2012a) showed OR-specific mistargeting and multiple ectopic glomeruli: MOR28 targeted correctly, but P2 and M71 did not. These three ORs project to different regions of the OB, indicating that the role of BACE1 in the precision of axonal targeting depends on the specific region of the OB (Cao et al., 2012a). The deletion of BACE1 also seemed to reduce TH levels in the OB compared to wild-type controls, again indicating reduced ORN input to the glomeruli.

This reduction in the input does not depend on neuronal loss in the OE, because in BACE1-KO mice the number of ORNs in the OE seems to be unaltered (Cao et al., 2012a). Intriguing is the idea that BACE1 has several other substrates besides APP (e.g., ephrin-A5 and protocadherins) that are cell-surface molecules with defined roles in axon guidance of mouse ORN axons (Cutforth et al., 2003; Hasegawa et al., 2008; Cao et al., 2012a). Whether BACE1 activity in the olfactory system is somehow connected to the fact that the olfactory system is extremely sensitive to AD progression is not clear yet, but it calls for careful evaluation when using an AD drug that targets BACE1 (Rajapaksha et al., 2011).

The observed axonal pathology might depend on the level of expression of BACE1 such that, when recruited for APP cleavage in animal models of AD, BACE1 no longer functions properly as a guidance/targeting receptor. Indeed, it has been suggested that changes in the expression pattern of proteins involved in homeostasis, within brain regions that express more of the proteins involved in APP processing, might make those regions more vulnerable to AD development (Freer et al., 2016). This could be the case of the olfactory system and its components (Rey et al., 2018). Apolipoprotein E is especially enriched in the primary olfactory pathway (Mahley, 1988; Nathan et al., 2007). In the OB, ApoE is expressed by glial cells around the glomeruli (Struble et al., 1999). Despite its expression in glial cells, ApoE is involved in controlling odor sensing and processing in mice (Zhang et al., 2018). In ApoE-KO animals, the entire OB circuitry is altered, indicating that ApoE may be responsible for the correct balance between excitatory and inhibitory inputs, which are altered in the OB of ApoE-KO mice because of reduced ORN signaling to the OB circuitry (Zhang et al., 2018). Altered OB circuitry, together with impaired olfactory-driven behavior, is also present in a mouse model carrying the humanized ApoE4 mutation (East et al., 2018).

## Conclusion and Future Perspectives

**FIGURE 3:**
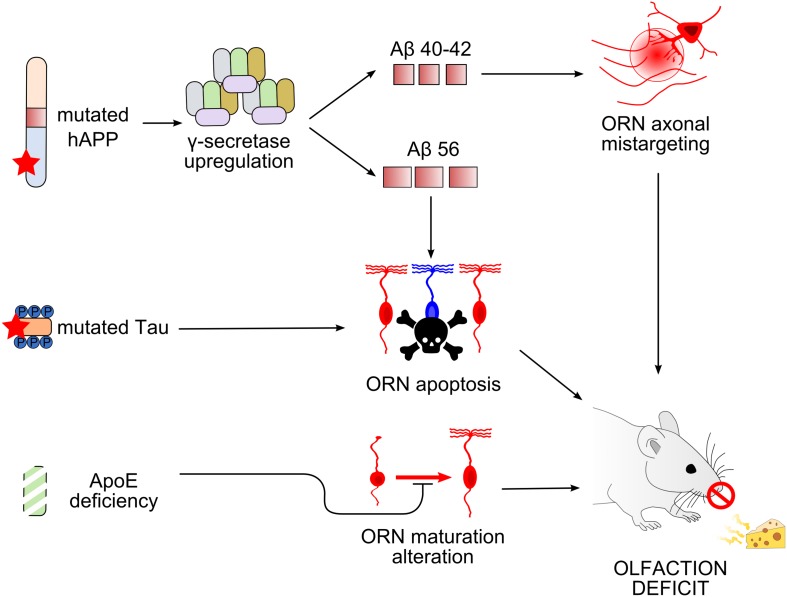
Working models of olfactory deficit arising in the olfactory epithelium in mouse models of Alzheimer’s disease. Top, Overexpression of mutated human amyloid precursor protein (hAPP) induces an upregulation of γ-secretase complex, leading to the production of different types of Aβ peptides (Cheng et al., 2011, 2016; Kim et al., 2018). Amyloid-β 40/42 (Aβ_40__/__42_) causes axonal mistargeting of olfactory receptor neurons (ORNs) in the olfactory bulb (OB) (Cao et al., 2012b), whereas Aβ_56_ causes ORN apoptosis (Kim et al., 2018). Middle, Mutation of tau also causes ORN apoptosis. Bottom, Apolipoprotein E deficiency alters the maturation of ORNs. All these alterations lead to deficits in olfactory-driven behaviors (Nathan et al., 2010; Zhang et al., 2018).

The sense of smell is impaired in patients suffering from AD and in individuals at risk of AD. This review has focused on AD pathophysiology relating to events taking place in the olfactory system at the early processing stages, in particular, the OE and OB in mouse models of AD (Figure 3). Similar to humans, mice show early AD pathophysiological symptoms in olfactory areas, both peripherally and centrally. Changes occurring in the OE that lead to neurodegeneration seem to be cell autonomous and independent of plaque accumulation.

Odorant responses in the OE are also decreased in several mouse models of AD, recapitulating the decreased olfactory ability observed in humans. In the OB, changes in AD pathophysiology involve mechanisms of glomerular map formation, altered circuitry, and neuroregeneration. The latter offers an interesting angle on future research to understand if olfactory regeneration can slow down or make the system more susceptible to olfactory decline. Tying in with this point, few studies have addressed the susceptibility of stem cells to AD-driven degeneration or why they no longer replace dying ORNs, which may warrant future studies, including how stem cells “burn out” in the elderly population at higher risk of AD and how well aging stem cells keep pace with neurodegeneration in AD mouse models. In particular, it would be important to understand how different mutations in AD mouse models drive different or similar olfactory AD symptoms. Interestingly, in addition to cells in the olfactory system, cells that express ORs outside the nose are differentially regulated in AD. Therefore, ORs, as GPCRs, could be a valid target for AD drug development, if their role in the brain, outside olfactory areas, and their differential regulation are confirmed and further understood.

The olfactory system seems highly vulnerable to AD, especially in its early stages. The OE may be a prime target to investigate hitherto elusive pathophysiological mechanisms that lead to AD. In this review, we have highlighted how this and other aspects of the olfactory system offer suitable models to address several unsolved mysteries of AD.

## Author Contributions

MD drafted the first version of the manuscript. All authors contributed to the manuscript revision.

## Conflict of Interest

The authors declare that the research was conducted in the absence of any commercial or financial relationships that could be construed as a potential conflict of interest.

## Abbreviations

A β: amyloid- β
AC3: adenylyl cyclase type 3
AD: Alzheimer’s disease
ApoE: apolipoprotein E
APP: amyloid precursor protein
BACE1: β-site amyloid precursor protein cleaving enzyme 1
GBCs: globose basal cells
GCs: granule cells
GPCRs: G protein–coupled receptors
hAPP: humanized APP
hAPPsw: naturally occurring Swedish human APP mutation
HBCs: horizontal or dark basal cells
KO: knockout
NFTs: neurofibrillary tangles
OB: olfactory bulb
OE: olfactory epithelium
OMP: olfactory marker protein
ORNs: olfactory receptor neurons
ORs: odorant receptors
PD: Parkinson’s disease
PGCs: periglomerular cells
PSEN1, PSEN2: secretase enzymes
SVZ: subventricular zone
TH: tyrosine hydroxylase.
